# Supplementary material for: Directional dependence of the plasmonic gain and nonreciprocity in drift-current biased graphene
Source: Nanophotonics. 2022 Nov 1;11(21):4929–36. doi: 10.1515/nanoph-2022-0451 (PMC11501735; doi:10.1515/nanoph-2022-0451)
Supplement: Supplementary file 1 — Supplementary Material Details [file j_nanoph-2022-0451_suppl.pdf]

Supplemental Material for the Manuscript

"Directional dependence of the plasmonic gain and nonreciprocity in drift-current biased graphene"

Tiago A. Morgado[1], Mário G. Silveirinha[2*]

[1]*Instituto de Telecomunicações and Department of Electrical Engineering, University of Coimbra, 3030-290 Coimbra, Portugal*

[2]*University of Lisbon–Instituto Superior Técnico and Instituto de Telecomunicações, Avenida Rovisco Pais, 1, 1049-001 Lisboa, Portugal*

E-mail: tiago.morgado@co.it.pt, mario.silveirinha@co.it.pt

In the supplementary note *A*) we present the surface conductivity tensor that characterizes the nonlocal intraband response of the bare graphene sheet. In the supplementary note *B*), we derive the reflection and transmission matrices for a two-dimensional (2D) material. The supplementary note *C*), derives the electromagnetic fields radiated by an electric dipole source nearby the 2D material. Finally, in supplementary note *D*), we present a more complete study of the graphene SPPs excited by emitters with different polarizations for the SiC substrate.

A. Surface conductivity tensor of a graphene sheet

The surface conductivity that characterizes an unbiased (i.e., without drifting electrons) graphene sheet can be written as:

$$\bar{\boldsymbol{\sigma}}_{\rm g} = \sigma_{\rm L}\left(\hat{\mathbf{k}}_{\rm t}\otimes\hat{\mathbf{k}}_{\rm t}\right)+\sigma_{\rm T}\left(\hat{\mathbf{z}}\times\hat{\mathbf{k}}_{\rm t}\right)\otimes\left(\hat{\mathbf{z}}\times\hat{\mathbf{k}}_{\rm t}\right), \tag{S1}$$

where $\mathbf{u}\otimes\mathbf{v}$ represents the tensor product of two generic vectors $\mathbf{u}$ and $\mathbf{v}$, $\hat{\mathbf{k}}_{\rm t}=\mathbf{k}_{\rm t}/\left|\mathbf{k}_{\rm t}\right|$ (with $\mathbf{k}_{\rm t}=k_x\hat{\mathbf{x}}+k_y\hat{\mathbf{y}}$ being the transverse wave vector), and $\sigma_{\rm L}$ and $\sigma_{\rm T}$ are the surface conductivities of the graphene sheet for longitudinal (with in-plane electric field parallel

---

[*] To whom correspondence should be addressed: E-mail: mario.silveirinha@co.it.pt

to the wave vector **k**) and transverse (with electric field perpendicular to the wave vector **k**) excitations, respectively.

Based on the theoretical model reported in the Appendix C of Ref. [1], the collisionless nonlocal longitudinal intraband conductivity of graphene is given by:

$$\sigma_{\mathrm{L}}(\omega,k)=\frac{i\omega e^2}{\hbar^2}\frac{2\mu_{\mathrm{c}}}{\pi}\frac{1}{\omega\sqrt{\omega-v_{\mathrm{F}}k}\sqrt{\omega+v_{\mathrm{F}}k}+\omega^2-v_{\mathrm{F}}^2k^2}, \tag{S2}$$

and the collisionless nonlocal transverse conductivity is given by

$$\begin{aligned}\sigma_{\mathrm{T}}(\omega,k)&=\frac{i\omega e^2}{\hbar^2}\frac{2\mu_{\mathrm{c}}}{\pi}\frac{\sqrt{\omega-v_{\mathrm{F}}k}\sqrt{\omega+v_{\mathrm{F}}k}}{\omega\left(\omega\sqrt{\omega-v_{\mathrm{F}}k}\sqrt{\omega+v_{\mathrm{F}}k}+\omega^2-v_{\mathrm{F}}^2k^2\right)}\\&=\frac{\sqrt{\omega-v_{\mathrm{F}}k}\sqrt{\omega+v_{\mathrm{F}}k}}{\omega}\sigma_{\mathrm{L}}(\omega,k)\end{aligned}, \tag{S3}$$

where ω is the oscillation frequency, e is the absolute value of the electron charge, $\hbar$ is the reduced Planck constant ($\hbar=h/(2\pi)$), μ_{c} is the chemical potential, $v_{\mathrm{F}}=c/300$ is the Fermi velocity, and $k=\sqrt{k_x^2+k_y^2}$.

The effect of collisions can be taken into account with the phenomenological correction [1]

$$\sigma_l^{\mathrm{col}}(\omega,k,\tau)=-i\omega\frac{\left(1+i/(\omega\tau)\right)\chi_l(\omega+i\tau,k)}{1+i/(\omega\tau)\chi_l(\omega+i\tau,k)/\chi_l(0,k)},\quad l=\mathrm{L,T}, \tag{S4}$$

where $\chi_l(\omega,k)=\sigma_l(\omega,k)/(-i\omega)$ is the susceptibility and τ is the relaxation time.

B. Reflection and transmission matrices

Here we obtain general formulas for the reflection and transmission matrices ($\overline{\mathbf{R}}$ and $\overline{\mathbf{T}}$) of a generic 2D material with an electromagnetic response characterized by a surface conductivity tensor (Fig. S1) in terms of admittance matrices.

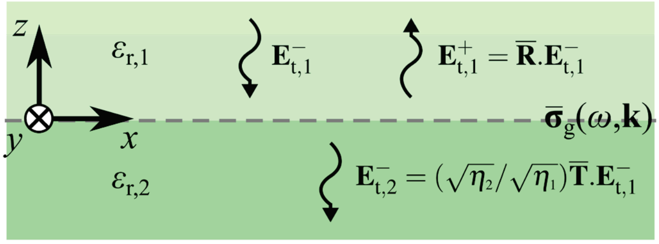

Fig. S1. Sketch of a graphene sheet surrounded by two dielectrics with relative permittivities $\varepsilon_{r,1}$ and $\varepsilon_{r,2}$ and wave impedances $\eta_1 = \eta_0/\sqrt{\varepsilon_{r,1}}$ and $\eta_2 = \eta_0/\sqrt{\varepsilon_{r,2}}$.

Following the approach of Refs. [2-3], we define the transverse fields as:

$$\mathbf{E}_t = \begin{pmatrix} E_x \\ E_y \end{pmatrix} \qquad \text{and} \qquad \bar{\mathbf{J}}\cdot\mathbf{H}_t = \begin{pmatrix} 0 & 1 \\ -1 & 0 \end{pmatrix}\begin{pmatrix} H_x \\ H_y \end{pmatrix} = \begin{pmatrix} H_y \\ -H_x \end{pmatrix}. \qquad \text{(S5)}$$

We introduce two admittance matrices $\bar{\mathbf{Y}}^{\pm}$ such that for plane waves propagating along the $+z$ and $-z$ directions one has:

$$\bar{\mathbf{J}}\cdot\mathbf{H}_t^+ = \bar{\mathbf{Y}}^+\cdot\mathbf{E}_t^+, \qquad \mathbf{J}\cdot\mathbf{H}_t^- = -\bar{\mathbf{Y}}^-\cdot\mathbf{E}_t^- \,. \qquad \text{(S6)}$$

The matrices $\bar{\mathbf{Y}}^{\pm}$ depend on the considered material, on the frequency ω, and on the transverse wave vector $\mathbf{k}_t$.

Let us consider the system illustrated in Fig. S1. By matching the tangential component of the electric field ($\mathbf{E}_{t,1}\big|_{z=0^+} - \mathbf{E}_{t,2}\big|_{z=0^-} = 0$) and by imposing the impedance boundary condition ($-\mathbf{J}.\left(\mathbf{H}_{t,1}\big|_{z=0^+} - \mathbf{H}_{t,2}\big|_{z=0^-}\right) = \bar{\boldsymbol{\sigma}}_g\cdot\mathbf{E}_t$) at the interface [1, 4], it is found that:

$$\left(\bar{\mathbf{1}}_t + \bar{\mathbf{R}}\right)\cdot\mathbf{E}_{1,t}^- = \mathbf{E}_{2,t}^-, \qquad \bar{\mathbf{Y}}_1^-\cdot\mathbf{E}_{t,1}^- - \bar{\mathbf{Y}}_1^+\cdot\mathbf{E}_{t,1}^+ - \bar{\mathbf{Y}}_2^-\cdot\mathbf{E}_{t,2}^- = \bar{\boldsymbol{\sigma}}_g\cdot\mathbf{E}_{t,2}^-. \qquad \text{(S7)}$$

where $\bar{\mathbf{1}}_t = \hat{\mathbf{x}}\otimes\hat{\mathbf{x}} + \hat{\mathbf{y}}\otimes\hat{\mathbf{y}}$ is the transverse identity matrix.

Since for isotropic media $\bar{\mathbf{Y}}_i = \bar{\mathbf{Y}}_i^{\pm}$ ($i = 1,2$), one obtains from (S7) that:

$$\bar{\mathbf{R}} = \left(\bar{\mathbf{Y}}_1 + \bar{\mathbf{Y}}_2 + \bar{\boldsymbol{\sigma}}_g\right)^{-1}\cdot\left(\bar{\mathbf{Y}}_1 - \bar{\mathbf{Y}}_2 - \bar{\boldsymbol{\sigma}}_g\right), \quad \text{and} \quad \bar{\mathbf{T}} = 2\sqrt{\frac{\eta_1}{\eta_2}}\left(\bar{\mathbf{Y}}_1 + \bar{\mathbf{Y}}_2 + \bar{\boldsymbol{\sigma}}_g\right)^{-1}\cdot\bar{\mathbf{Y}}_1 \qquad \text{(S8)}$$

The admittance matrices $\mathbf{Y}_i$ ($i = 1,2$) are given by [2-3]:

$$\mathbf{Y}_i = \frac{1}{\eta_0 k_0 k_{z,i}^+}\begin{bmatrix} k_0^2\varepsilon_{\mathrm{r},i} - k_y^2 & k_x k_y \\ k_x k_y & k_0^2\varepsilon_{\mathrm{r},i} - k_x^2 \end{bmatrix}, \tag{S9}$$

where $k_{z,i}^+ = \sqrt{k_0^2\varepsilon_{\mathrm{r},i} - k_x^2 - k_y^2}$, $k_0 = \omega\sqrt{\varepsilon_0\mu_0}$ is the free-space wave number, and $\eta_0 = \sqrt{\mu_0/\varepsilon_0} = 120\pi\ [\Omega]$ is the free-space impedance.

The same result can be derived in a different way, as follows. Specifically, the reflection matrix (in the absence of a drift current) can be written as $\mathbf{R} = R_{\mathrm{TM}}\left(\hat{\mathbf{k}}_\mathrm{t} \otimes \hat{\mathbf{k}}_\mathrm{t}\right) + R_{\mathrm{TE}}\left(\hat{\mathbf{z}} \times \hat{\mathbf{k}}_\mathrm{t}\right) \otimes \left(\hat{\mathbf{z}} \times \hat{\mathbf{k}}_\mathrm{t}\right)$, with R_{TM} and R_{TE} the scalar electric reflection coefficients of the graphene sheet for plane waves incident with transverse magnetic (TM) and electric (TE) polarization. This decomposition is valid provided the surface conductivity has the structure $\bar{\boldsymbol{\sigma}}_\mathrm{g} = \sigma_\mathrm{L}\left(\hat{\mathbf{k}}_\mathrm{t} \otimes \hat{\mathbf{k}}_\mathrm{t}\right) + \sigma_\mathrm{T}\left(\hat{\mathbf{z}} \times \hat{\mathbf{k}}_\mathrm{t}\right) \otimes \left(\hat{\mathbf{z}} \times \hat{\mathbf{k}}_\mathrm{t}\right)$

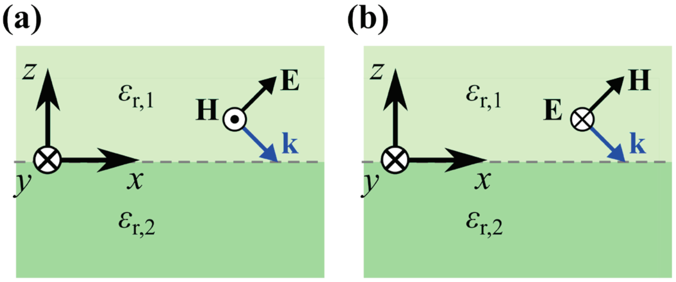

Fig. S2. Sketch of a graphene sheet illuminated by (a) a TM polarized wave and (b) a TE polarized wave.

Thus, R_{TM} and R_{TE} are coincident with the reflection coefficients for TM and TE polarized waves, for a plane wave illuminating an equivalent isotropic 2D material with surface conductivity σ_L and σ_T, respectively. This observation implies that:

$$R_{\mathrm{TE}}\left(\omega, k_x, k_y\right) = \frac{\gamma_1 - \gamma_2 + ik_0\eta_0\sigma_\mathrm{T}}{\gamma_1 + \gamma_2 - ik_0\eta_0\sigma_\mathrm{T}}$$

$$R_{\mathrm{TM}}\left(\omega, k_x, k_y\right) = \frac{\gamma_2\varepsilon_{\mathrm{r},1} - \gamma_1\varepsilon_{\mathrm{r},2} + \gamma_1\gamma_2 \dfrac{\sigma_\mathrm{L}}{i\omega\varepsilon_0}}{\gamma_2\varepsilon_{\mathrm{r},1} + \gamma_1\varepsilon_{\mathrm{r},2} - \gamma_1\gamma_2 \dfrac{\sigma_\mathrm{L}}{i\omega\varepsilon_0}}, \tag{S10}$$

$\gamma_1 = \sqrt{k_x^2 + k_y^2 - \varepsilon_{\mathrm{r},1}(\omega / c)^2}$ and $\gamma_2 = \sqrt{k_x^2 + k_y^2 - \varepsilon_{\mathrm{r},2}(\omega / c)^2}$ are the propagation constants along z in regions 1 and 2, respectively. It can be checked that the above formulas lead to an $\mathbf{R}$ that agrees exactly with Eq. S8.

C. Fields radiated by an electric dipole

Here, we present the formulas for the electric field radiated by a Hertz electric dipole nearby a 2D material (e.g., a graphene sheet) [see Fig. S3]. The radiated and scattered fields are written in terms of Sommerfeld-type integrals.

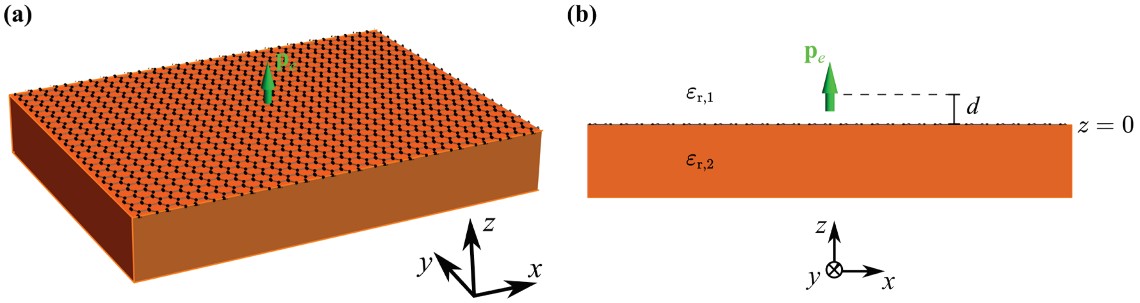

Fig. S3. An electric dipole stands above a graphene sheet. (a) Perspective view; (b) Front view.

The electromagnetic field in the region $z > 0$ where is located the dipole is given by the superposition of the primary field (i.e., the field radiated by the dipole) and the scattered field (i.e., the field scattered by the graphene sheet). The primary or radiated electric field is given by

$$\begin{aligned}
\mathbf{E}^{\mathrm{inc}} &= \left(\nabla\nabla + k_0^2 \varepsilon_{\mathrm{r},1} \overline{\mathbf{1}}\right)\Phi_0 \cdot \frac{\mathbf{p}_e}{\varepsilon_0 \varepsilon_{\mathrm{r},1}} \\
\Phi_0 &= \frac{e^{ik_0\sqrt{\varepsilon_{\mathrm{r},1}}|\mathbf{r}-\mathbf{r}'|}}{4\pi|\mathbf{r}-\mathbf{r}'|} = \frac{1}{(2\pi)^2}\iint dk_x dk_y \frac{e^{-\gamma_1|z-d|}}{2\gamma_1} e^{i(k_x x + k_y y)}
\end{aligned}, \tag{S11}$$

Following the formalism of Ref. [5] and of the Appendix B of Ref. [6], the scattered electric field above the graphene sheet (i.e., the electric field reflected by the graphene sheet) can be written as:

$$\mathbf{E}^{\mathrm{ref}} = \frac{1}{(2\pi)^2}\iint d^2\mathbf{k}_{\mathrm{t}} \frac{e^{-\gamma_1(z+d)}}{2\gamma_1} e^{i\mathbf{k}_{\mathrm{t}}\cdot\mathbf{r}} \mathbf{C}_1(\omega, \mathbf{k}_{\mathrm{t}}) \cdot \frac{\mathbf{p}_e}{\varepsilon_0 \varepsilon_{\mathrm{r},1}}, \tag{S12}$$

where

$$\mathbf{C}_1(\omega,\mathbf{k}_t)=\left[\overline{\mathbf{1}}_t+\hat{\mathbf{z}}\frac{i\mathbf{k}_t}{\gamma_1}\right]\cdot\overline{\mathbf{R}}(\omega,\mathbf{k}_t)\cdot\left[i\gamma_1\mathbf{k}_t\hat{\mathbf{z}}+k_0^2\varepsilon_{r,1}\overline{\mathbf{1}}_t-\mathbf{k}_t\mathbf{k}_t\right], \tag{S13}$$

$\overline{\mathbf{1}}_t=\hat{\mathbf{x}}\hat{\mathbf{x}}+\hat{\mathbf{y}}\hat{\mathbf{y}}$ is the transverse identity matrix, $\mathbf{k}_t=k_x\hat{\mathbf{x}}+k_y\hat{\mathbf{y}}$ is the transverse wave vector, $\gamma_1=-ik_{z,1}=\sqrt{k_x^2+k_y^2-\omega^2\varepsilon_0\varepsilon_{r,1}\mu_0}$ is the propagation constant along z in region 1 ($z>0$), and $\overline{\mathbf{R}}$ is the reflection matrix that relates the tangential (to the interface) components x and y of the reflected electric field to the corresponding x and y components of the incident field, $\begin{pmatrix}E_x^{\text{ref}}\\E_y^{\text{ref}}\end{pmatrix}=\overline{\mathbf{R}}(\omega,\mathbf{k}_t)\cdot\begin{pmatrix}E_x^{\text{inc}}\\E_y^{\text{inc}}\end{pmatrix}$, for the case of plane wave incidence.

On the other hand, the scattered electric field below the graphene sheet (i.e., the electric field transmitted through the graphene sheet) can be written as:

$$\mathbf{E}^{\text{tr}}=\frac{1}{(2\pi)^2}\iint\frac{1}{2\gamma_1}e^{-\gamma_1 d}e^{\gamma_2 z}e^{i\mathbf{k}_t\cdot\mathbf{r}}\mathbf{C}_2(\omega,\mathbf{k}_t).\frac{\mathbf{p}_e}{\varepsilon_0\varepsilon_{r,1}}d^2\mathbf{k}_t\,, \tag{S14}$$

where

$$\mathbf{C}_2(\omega,\mathbf{k}_t)=\left[\overline{\mathbf{1}}_t-\hat{\mathbf{z}}\frac{i\mathbf{k}_t}{\gamma_2}\right]\cdot\sqrt{\frac{\eta_2}{\eta_1}}\overline{\mathbf{T}}(\omega,\mathbf{k}_t)\cdot\left[i\gamma_1\mathbf{k}_t\hat{\mathbf{z}}+k_0^2\varepsilon_{r,1}\overline{\mathbf{1}}_t-\mathbf{k}_t\mathbf{k}_t\right], \tag{S15}$$

and $\gamma_2=-ik_{z,2}=\sqrt{k_x^2+k_y^2-\omega^2\varepsilon_0\varepsilon_{r,2}\mu_0}$ is the propagation constant along z in region 2 ($z<0$), and $\overline{\mathbf{T}}$ is the transmission matrix that relates the tangential (to the interface) components x and y of the transmitted electric field to the corresponding x and y components of the incident field, $\begin{pmatrix}E_x^{\text{tr}}\\E_y^{\text{tr}}\end{pmatrix}=\sqrt{\frac{\eta_2}{\eta_1}}\overline{\mathbf{T}}(\omega,\mathbf{k}_t)\cdot\begin{pmatrix}E_x^{\text{inc}}\\E_y^{\text{inc}}\end{pmatrix}$, for the case of plane wave incidence.

In the Galilean model, the reflection and transmission matrices in the presence of a drift-current bias along the x-direction are related to the no-drift matrices derived in Sect. A as $\tilde{\mathbf{R}}(\omega, k_x, k_y) = \overline{\mathbf{R}}(\tilde{\omega}, k_x, k_y)$ and $\tilde{\mathbf{T}}(\omega, k_x, k_y) = \overline{\mathbf{T}}(\tilde{\omega}, k_x, k_y)$, where $\tilde{\omega} = \omega - k_x v_0$ is the Doppler-shifted frequency and v_0 is the drift velocity of the electrons on the graphene sheet along the x-direction.

D. Detailed study of the SPPs excited by an electric dipole emitter

Figure S4 present time snapshots of the SPPs propagating on a drift-current biased graphene deposited on a SiC substrate (analogous to Fig. 5(a) of the main text) and excited by short electric dipoles with different dipole moments $\mathbf{p}_e$.

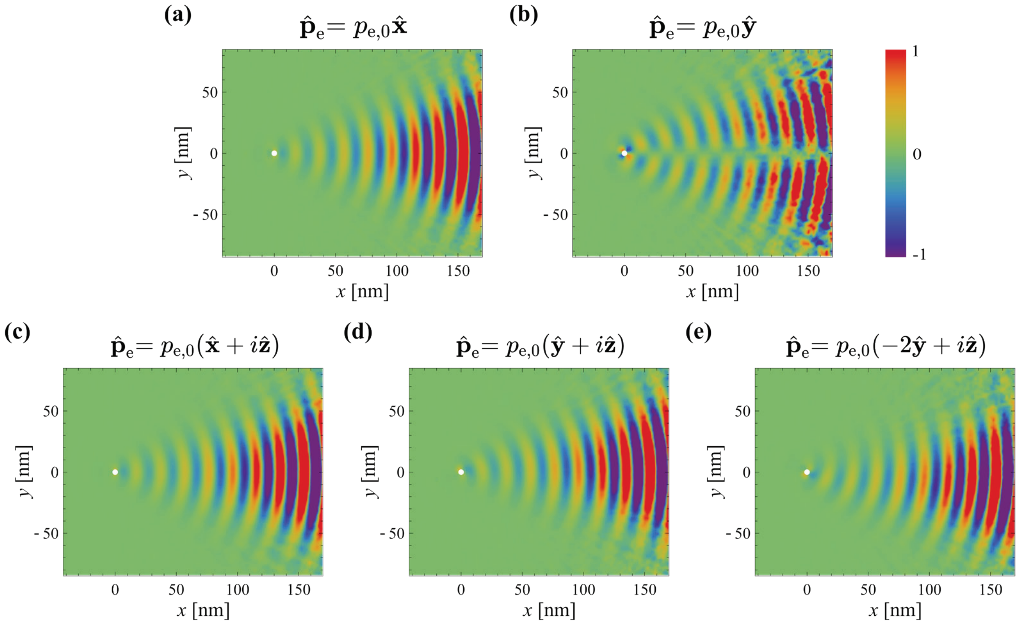

Fig. S4. (a-e) Time snapshots of the x-component of the electric field E_x (in arbitrary unities) on the surface of the graphene sheet for electric dipoles placed 1 nm above the interface, calculated using the Galilean model with $v_0 = 0.6 v_F$. The electric dipole moment of each dipole is indicated on the top of each panel. In all the panels the frequency of operation is $\omega/(2\pi) = 25$ THz, $\mu_c = 0.1$ eV, $\tau = 1.7$ ps, $\varepsilon_{r,1} = 1$, and $\varepsilon_{r,2} = \varepsilon_{r,Sic}$.

Figure S4 shows that the graphene-SiC system enables SPP amplification for linearly polarized horizontal dipoles, as well as for dipoles with circular and elliptical

polarizations, similar to that shown in Fig. 5(a) of the main text for a linearly polarized vertical dipole.

REFERENCES

1. P. A. D. Gonçalves, N. M. R. Peres, *An introduction to graphene plasmonics,* World Scientific, Hackensack, NJ, 2016.
2. T. A. Morgado and M. G. Silveirinha, "Single-interface Casimir torque", *New J. Phys.* **18**, 103030 (2016).
3. H. Latioui and M. G. Silveirinha, "Lateral optical forces on linearly polarized emitters near a reciprocal substrate," *Phys. Rev. A* **100**, 053848 (2019).
4. G. W. Hanson, "Dyadic Green's functions and guided surface waves for a surface conductivity model of graphene," *J. Appl. Phys.* **103**, 064302 (2008).
5. M. G. Silveirinha, "Optical Instabilities and Spontaneous Light Emission by Polarizable Moving Matter," *Phys. Rev. X* **4**, 031013 (2014).
6. M. G. Silveirinha, S. A. H. Gangaraj, G. W. Hanson, and M. Antezza, "Fluctuation-induced forces on an atom near a photonic topological material," *Phys. Rev. A* **97**, 022509 (2018).
